# Study on the Effect of Processing Parameters on Residual Stresses of Injection Molded Micro-Pillar Array

**DOI:** 10.3390/polym14163358

**Published:** 2022-08-17

**Authors:** Xiaoyu Zhang, Tao Ding, Wanlin Wang, Jiezhen Liu, Can Weng

**Affiliations:** College of Mechanical and Electrical Engineering, Central South University, Changsha 410083, China

**Keywords:** residual stress, injection molding, micro-pillar array, processing parameter

## Abstract

As one of the main methods for fabricating microstructured surfaces, micro-injection molding has the advantages of short cycle time, high production efficiency, and the potential for batch manufacturing. However, non-negligible residual stresses inside the molded part could affect the replication quality, dimensions, and physical properties of the microstructure. Therefore, studying the effects of processing parameters on residual stresses is a necessary prerequisite to ensure the successful fabrication of microstructured parts. In this paper, an injection molding simulation model of micro-pillar arrays was developed using molecular dynamics software, and a series of injection molding experiments were conducted. It was found that increasing the mold temperature and melt temperature can reduce the thermal residual stresses and molecular orientation stresses, and effectively improve the uniformity of residual stress distribution. The increase in the packing pressure can make the shear field of flow more intense and increase the molecular orientation stresses, thus making the residual stresses more severe.

## 1. Introduction

Due to the rapid development of micro/nano processing technology, it has been widely used in the fabrication of micro/nanostructured devices. The miniaturization, complexity, intelligence, and integration of the components have gradually become the goal that researchers are constantly pursuing. The functional surface with micro/nanostructures is a surface where micro/nano single-scale or multi-scale features form regular textures, periodic arrangements, and different functional properties through different sizes and distributions [1]. Therefore, it is increasingly used in microfluidic chips [2,3], conversion surfaces [4], biomimetic surfaces [5], and superhydrophobic surfaces [6,7]. Due to the advantages of short cycle time, high production efficiency, and batch manufacturing, micro-injection molding technology has been increasingly used in the fabrication of micro/nanostructured surfaces. Different temperatures or pressures in injection molding cause the melt to experience different cooling rates and shear field intensities [8,9,10], resulting in residual stresses that are difficult to eliminate. The existence of residual stresses will directly affect the mechanical and optical properties of the part, and in serious cases will cause warping and cracking. Additionally, it will greatly affect the replication quality of micro/nanostructures, such as deformation, bending, and loosening of micro/nanostructures. Therefore, it is important to explore the molding mechanism and influence mechanism of residual stresses in the micro-injection molding process for the high-quality molding of polymer micro/nanostructures.

Scholars have conducted a lot of research on two aspects through numerical simulations and experimental studies. R. T. Tebeta et al. [11] explored the effect of single-walled carbon nanotubes (SWCNTs) reinforced materials on the elastic modulus of high-density polyethylene (HDPE) using experiments and the finite element method. The results showed that the elastic modulus and yield stress of HDPE were improved by adding SWCNT nanoparticles, and HDPE/SWCNTs nanocomposites with higher elastic modulus were obtained by injection molding compared with compression molding. Xie et al. [12] developed a dynamic visualization system for injection molding, and they believed that the larger the gate, the faster the rate of melt filling the cavity, and the smaller the residual stresses of the part. Li et al. [13] used orthogonal experiments to study the effect of different processing parameters on the residual stresses of polyoxymethylene (POM) gears and reduced the stresses through parameter optimization. Jansen et al. [14] pointed out by means of numerical calculation and experimental comparison that local cavity pressure was considered to be the most important factor affecting residual stresses, warpage, and shrinkage. Most of the current studies on residual stress formation in polymer injection molding are based on continuum simulations. Molecular dynamics (MD) simulation, a computer simulation method to study the physical motion and interaction between molecules, has been widely used in injection molding to analyze the molding quality [9,15,16,17], geometry, and even the filling behaviors of polymers. Yang et al. [18] studied polymethyl methacrylate (PMMA) nano-imprint molding using molecular dynamics simulation and pointed out that there was some difference in the potential energy of the PMMA at the beginning and the end of imprinting. The deformation was not completely released, and residual stresses still existed. The higher the melt temperature, the greater the potential energy difference. Kang et al. [19] used molecular dynamics simulations to investigate the deformation behaviors of PMMA during nano-imprinting and compared the stress distribution of the polymer under different aspect ratios of SiO_2_ molds. The study also pointed out that when the imprint separation occurred, there would be larger residual compressive stresses on the surface of the nanostructures with a larger aspect ratio.

The purpose of this paper is to explore the mechanism of the effects of processing parameters on residual stresses of micro-pillar arrays. A model of polypropylene (PP) filling in the injection molding process was established using the MD method. The changes in residual stress distribution, melt density, and molecular orientation angle in the cavity with different processing parameters were analyzed. The effects of mold temperature, melt temperature, and packing pressure on residual stresses in micro-pillar arrays were further studied by comparing with the experimental results of injection molding.

## 2. Micro-Injection Molding Experiments

### 2.1. Materials and Injection Molding Process

The design of micro-pillar array and the fabrication of its nickel (Ni) mold insert were described in detail in our previous paper [20]. The average dimensions of the designed micro-pillar array, its Si master and the Ni mold insert are listed in Table 1.

Considering the transparency of the material required for the subsequent birefringence measurement and the difficulty of molding micro-pillar arrays, Taiwan Formosa Plastics PP-5090T material was selected for the experiments, and its properties are listed in Table 2. An electrical injection molding machine (LD05EH2, Sodick®, Yokohama, Japan) was used for injection molding experiments.

Table 3 lists the processing parameters used in the experiments. To ensure the accuracy of the measurements, samples were taken from the sixth product of each set of processing parameters.

### 2.2. Morphological Characterization

The three-dimensional morphology and contour of micro-pillar arrays were measured with a digital microscope (VHX-500, Keyence®, Osaka, Japan). The residual stress distributions of the molded parts were examined by a polarized stress tester (WPA-200, Photonic lattic®, Minami-Yoshinari, Japan), and the three-dimensional topographies of the demolded micro-pillar arrays were detected with a laser confocal microscope (LSM 700, Zeiss® corp, Oberkochen, Germany).

## 3. Molecular Dynamics Simulation

### 3.1. Model Construction

**Figure 1:**
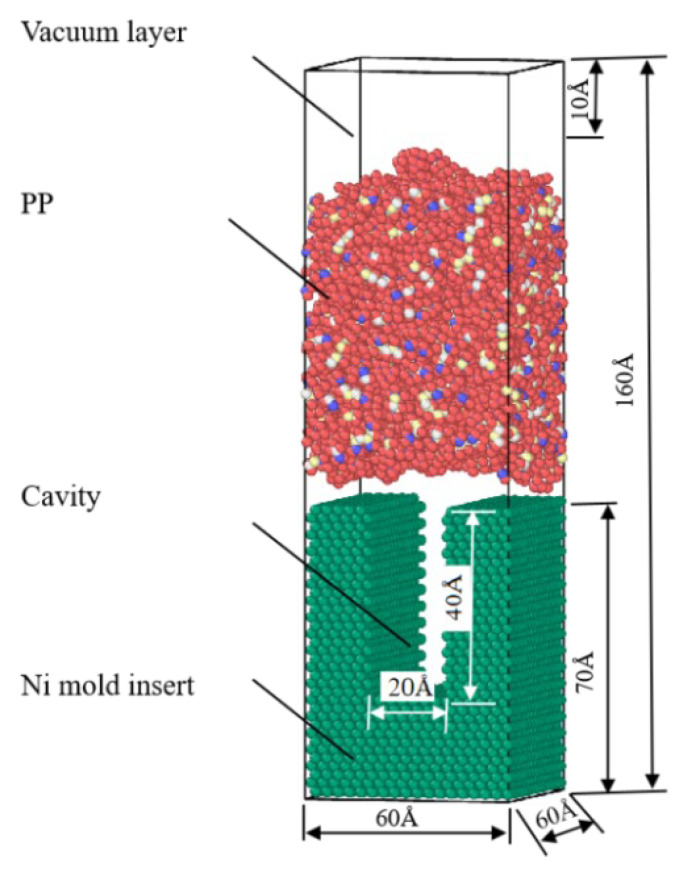
The polymer-melt molecular model for the injection molding process.

A simulation model was built using Materials Studio 7.0. A vacuum layer with a depth of 10 Å was added above the model to ensure the accuracy of the simulation results. An amorphous model of PP polymer was established in a cube of 60 Å × 60 Å × 160 Å with a polymerization degree of 50. The cut-off radius of the single-chain PCFF force field was set to 12.5 Å, the temperature to 298 K, the density to 0.90 g/cm^3^, and the number of chains to 20. After importing the pure metal Ni cell, a cell with a height of 70 Å was cut on the (1 1 0) crystal plane, and a cavity with a width of 20 Å and a depth of 40 Å was constructed by deleting some nickel molecules. Finally, the established polymer–metal model was shown in Figure 1.

### 3.2. Simulation Procedure

**Figure 2:**
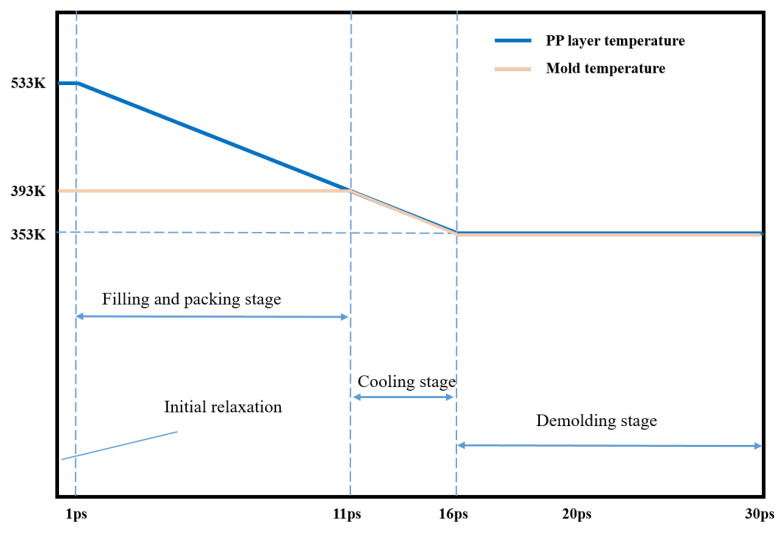
Time and temperature settings of simulation procedure for the injection molding process.

The simulations were performed using a large-scale atomic/molecular massively parallel simulator (LAMMPS), which was an open-source molecular dynamics package in a computer cluster. In the simulation, the X and Y directions were set as fixed boundary conditions, the Z direction was set as free boundary conditions. Considering that the polymer melt contacts the cavity wall and solidifies rapidly in the simulation, and the melt volume is almost negligible relative to the Ni mold insert, to simplify the simulation, the mold temperature is regarded as the temperature of the system, and the NVT ensemble is selected as the ensemble of the system in the simulation. A force of 1.0 kcal/mol Å (equal to 0.07 nN) was applied to each PMMA atom during the filling and packing process so that the polymer can fill the nano-cavity smoothly. The simulation procedure for the injection molding process included filling, packing, cooling, and the temperature of the injection molding process was set as shown in Figure 2. The PP layer was first heated to the molten state at the temperature of 513 K and relaxed for 1 ps at a constant set of particle number, volume, and temperature. In the next filling process, the mold temperature was set as 393 K to make the polymer melt temperature drop to it, and a constant particle number, volume, and energy ensemble were used to keep the total energy of the system constant. Self-heating conduction between the PP layer and the nickel mold insert was added. This is followed by the cooling stage, in which the melt and the mold are cooled down to 353 K. Next, under a demolding force of 1.0 kcal/mol Å in the opposite direction, the formed structure moved upward and finally out of the cavity. In addition, since Young’s modulus of PP is negligible compared with that of Ni, so all Ni atoms were fixed in their initial positions. In the whole simulation process, the time step, and the total step number were 0.2 fs, and 150,000, respectively.

## 4. Results and Discussion

### 4.1. Effect of the Mold Temperature on Residual Stresses

**Figure 3:**
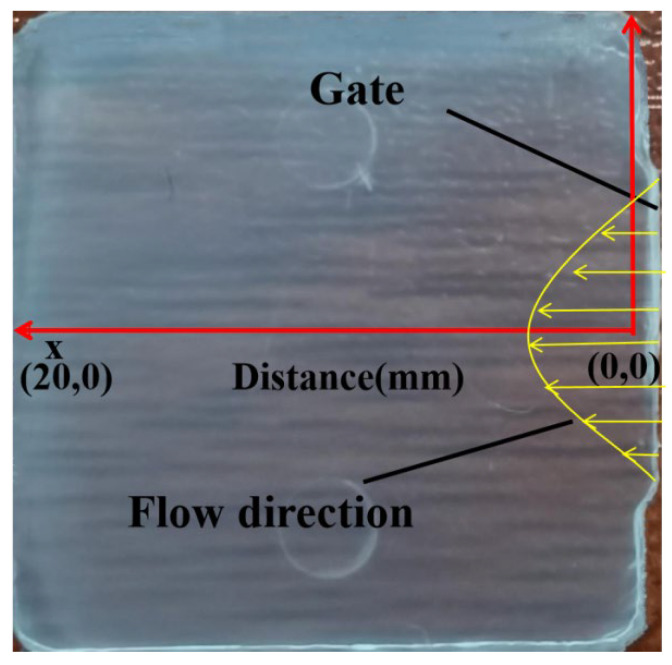
Physical drawing of the part.

The horizontal and vertical midpoint position on the molded part is the origin, and the centerline is the horizontal axis as shown in Figure 3. Additionally, the position of the gate and flow direction is marked on the part.

**Figure 4:**
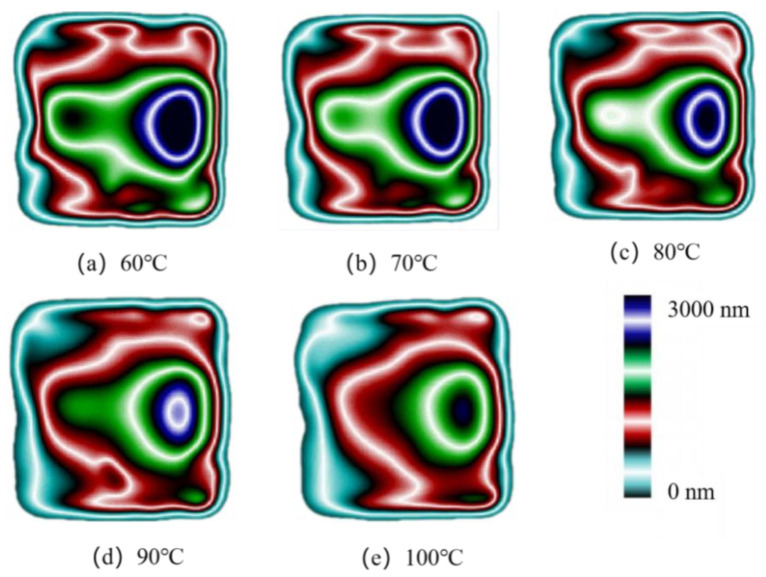
Birefringence distributions at different mold temperatures.

Figure 4 shows the birefringence distribution of residual stresses in the molded parts at different mold temperatures after injection molding in the experimental study. It can be seen that the overall distribution of residual stresses is axisymmetric along the centerline of the transverse axis and the further away from the gate, the smaller the stress value. Four different color stress zones are shown in the figure, corresponding to the stresses from high to low: dark blue, green, red, and light blue. The dark blue layer area has the highest stress value and forms an ellipse. By comparison, it can be clearly seen that the area of the dark blue layer and the green layer near the gate reduces with the increase in the mold temperature. When the mold temperature increases to 100 °C, the area of the dark blue layer basically disappears, while the area of the green layer on the left also disappears. Meanwhile, the outermost blue layer and the red layer keep expanding. It can be seen that the uniformity of residual stress distribution is effectively improved when the mold temperature increases.

Based on the optical path difference output in Figure 4, the residual stresses of molded parts can be calculated according to the optical stress theorem, as shown in Equation (1):

(1) $$\sigma =\frac{\Delta S}{K\times h}$$

where σ represents residual stress value, (MPa); ΔS represents optical path difference, (nm); K represents the pressure optical coefficient, h represents the thickness of the part being measured, (mm). According to the literature, the strain optical coefficient of PP is 1.30 × 10^−10^ Pa^−1^ [21], residual stresses then can be calculated.

**Figure 5:**
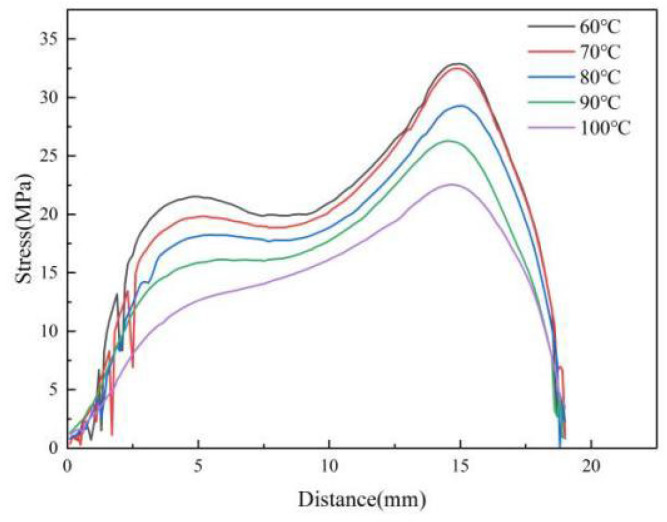
Variations of stress curve at different mold temperatures along the centerline of the parts.

**Figure 6:**
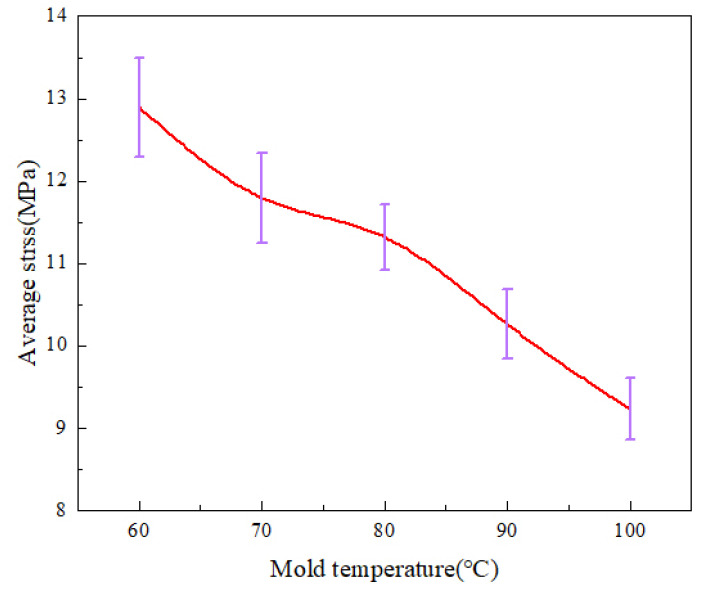
Average stress variations at different mold temperatures along the center line of the parts.

Figure 5 shows the stress variation curves along the centerline of the parts by calculation at various mold temperatures. As the mold temperature grows, the stress values of the parts decrease significantly. It can also be seen that the curves become smoother as the temperature increases and the stress concentration zone disappears at 5 mm from the origin, indicating that the uniformity of residual stresses is improved. Figure 6 shows the average stress variations at different mold temperatures. When the mold temperature increases from 60 °C to 100 °C, the average stress decreases from 12.9 MPa to 9.2 Mpa, a decrease of 28.4%. The average stresses decrease significantly when the mold temperature increases.

**Figure 7:**
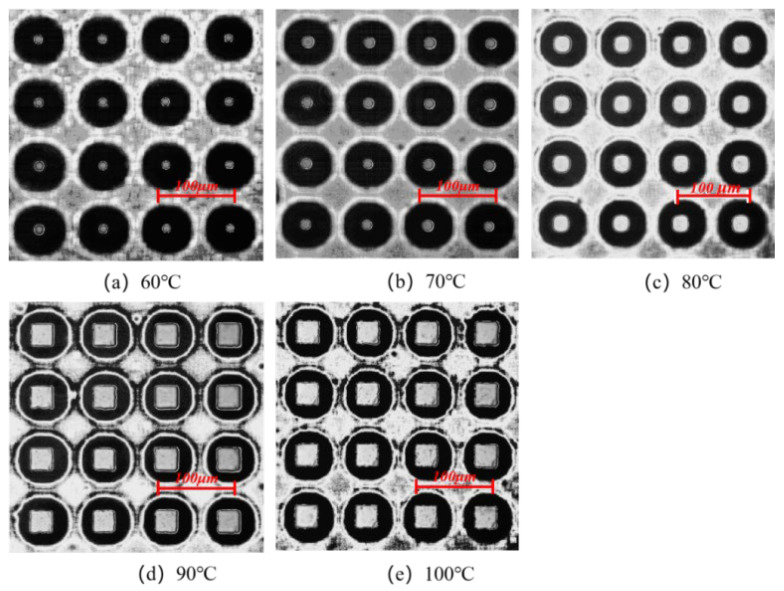
Microstructure replication morphologies at different mold temperatures.

The molding morphologies of the micro-pillar arrays at different mold temperatures in the experimental study are shown in Figure 7. When the mold temperatures are 60 °C and 70 °C, respectively, the microstructures are presented only in the middle part with poor molding quality. When the temperature increases to 90 °C, it can be seen that the microstructures are filled completely without obvious defects. This phenomenon indicates that the higher the mold temperature, the smaller the shear effect and the lower the stress in the cavity before demolding, thus ensuring a smooth demolding stage. In addition, a higher mold temperature allows for slower melt cooling and sufficient time for filling. Therefore, increasing the mold temperature not only improves the residual stresses in the part after demolding, but also improves the quality of the microstructures.

**Figure 8:**
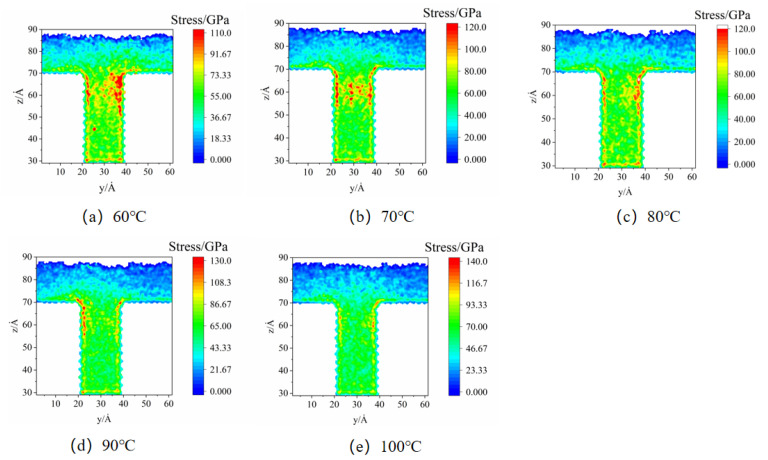
Stress distributions at different mold temperatures in MD simulation.

The stress distributions of nanostructures at different mold temperatures in the MD simulation are illustrated in Figure 8. It is found that the stress concentration phenomenon is particularly severe at the mold temperature of 60 °C, especially in the middle of the cavity. As the mold temperature keeps increasing, the stress concentration phenomenon begins to weaken, and the stress concentration region in the middle of the cavity gradually disappears. When the mold temperatures are 80 °C and 90 °C, respectively, the stress concentration areas are mainly distributed at the edges of the cavity. This may be the critical temperature for the formation of the condensation layer. As the temperature difference between the melt and the mold becomes larger, the thickness of the condensation layer becomes larger. When the mold temperature increases to 100 °C, the stress distribution in the cavity is relatively uniform, which is consistent with the experimentally observed phenomenon. It can be noticed that in the simulation, the stress is at the GPa level, while in the experiment it is at the MPa level. This is because the fact that microstructures are used in experiments, but nanostructures are used in the simulations. Additionally, stress is the ratio of pressure to the area, so the difference in stress values is several orders of magnitude.

**Figure 9:**
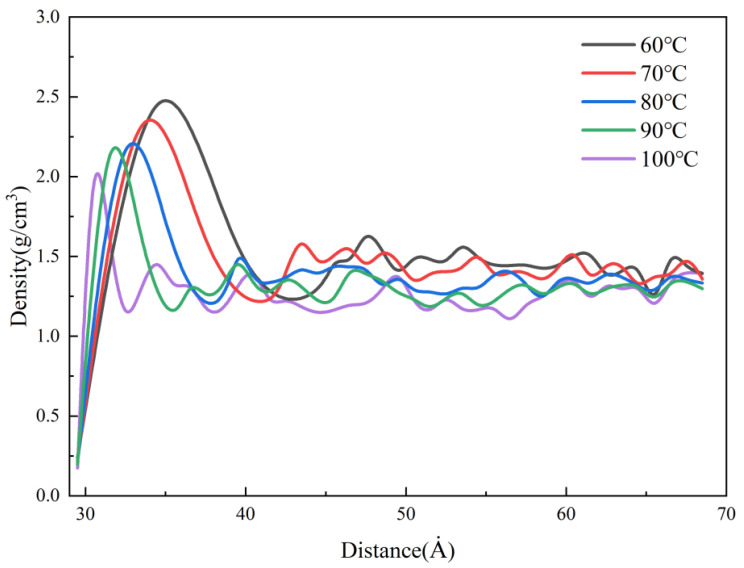
Density distributions at different mold temperatures along the flow direction.

The density distributions in the cavity at different mold temperatures in MD simulation are presented in Figure 9. The stable values of density in the cavity, the density values, and the thicknesses of the condensation layer are increasing with the decrease in the mold temperature. The melt cooling rate is defined as Equation (2).

(2) $$v=\frac{T_{1}-T_{2}}{t}$$

where T_1_ is the initial melt temperature, 533 K, and T_2_ is the melt temperature during filling and cooling process, which is affected by the mold temperature, (K); t is melt cooling time, (ps) which is set to a constant value in the simulation. This phenomenon indicates that the larger the temperature difference between the polymer and the mold, the faster the melt cooling rate and the greater the shrinkage of the polymer. The magnitude of thermal residual stress is related to the melt cooling rate. As the melt cooling rate increases, the value of thermal residual stress increases. So, when the mold temperature is lower, the polymer needs a longer cooling time, which leads to an increase in the thickness of the condensation layer. When the mold temperature is 60 °C, the thickness of the condensation layer is about 10 Å, while at a mold temperature of 100 °C is only 2 Å. In the experiments, the higher mold temperature results in slower cooling of the melt and lower thermal stresses to allow enough time for filling, thus improving the replication of microstructures and reducing residual stresses in the parts, as shown in Figure 5 and Figure 7.

**Figure 10:**
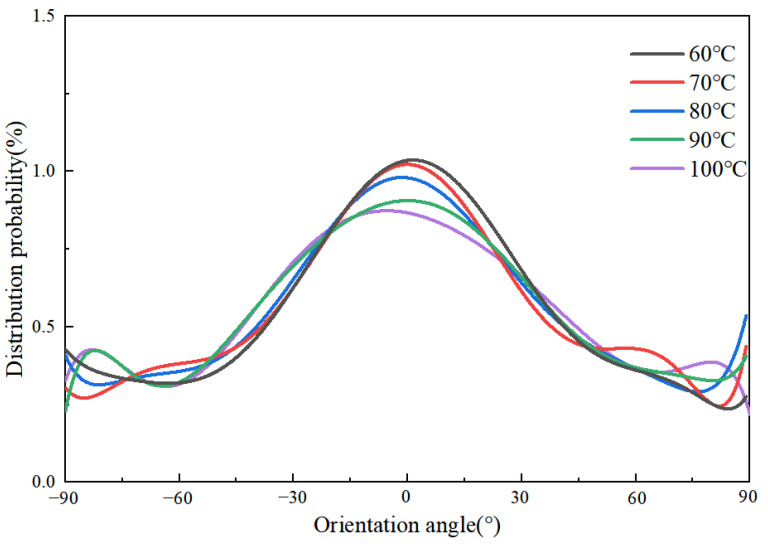
Orientation angle distributions of PP at different mold temperatures along the flow direction.

Figure 10 shows the variations in the orientation angle of PP for different mold temperatures in the MD simulation. As the mold temperature increases, the orientation angle along the flow direction decreases, and its peak values near 0° also decreases. This is because the viscosity of the filled polymer increases as it gradually cools to the mold temperature. Therefore, when the mold temperature is lower, the flow resistance of the PP melt is higher, and the orientation stress is correspondingly larger. This phenomenon indicates that the mold temperature will affect the orientation stress. The weaker the shear effect, and the lower the stresses in the cavity before demolding, thus ensuring a smooth demolding stage and improving the residual stresses in the parts and the replication quality of microstructures. By increasing the mold temperature, not only the residual stresses will be reduced, but also the uniformity of distribution also will be improved.

### 4.2. Effect of Packing Pressure on Residual Stresses

**Figure 11:**
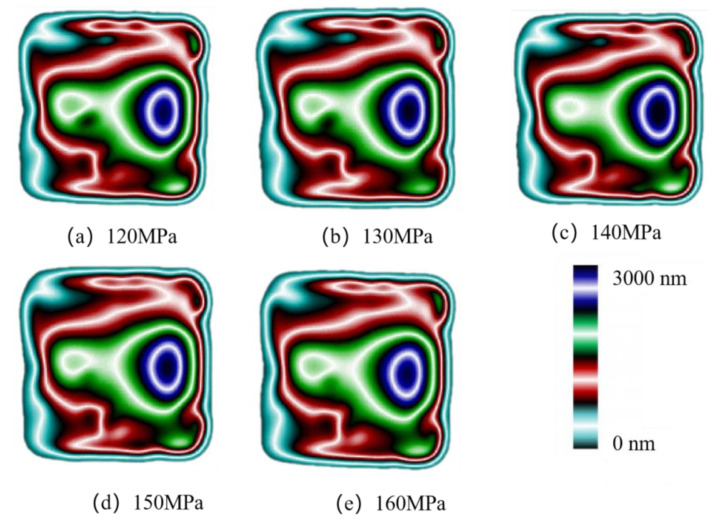
Birefringence distributions at different packing pressures.

**Figure 12:**
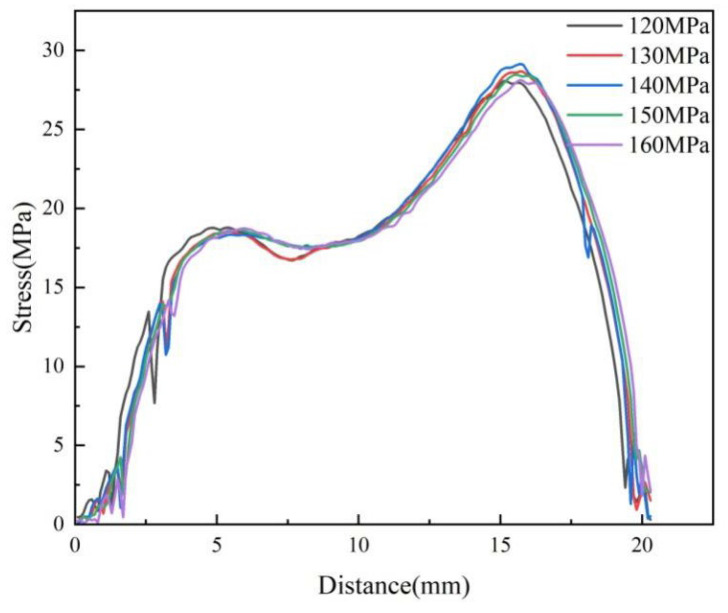
Variations of stress curve at different packing pressures along the centerline of the parts.

In injection molding, the packing pressure mainly plays a role in making the melt completely fill the cavity, and it has the most direct effect on the flow shear field. In the experimental study, the birefringence distribution of residual stresses in the molded parts with different packing pressures in the experimental study is shown in Figure 11. It can be found that the distribution of residual stresses remains basically constant with the increase in the packing pressure. The stresses are calculated according to Equation (1) as shown in Figure 12. The peak stress increases from 28.1 MPa to 29.3 MPa when the packing pressure increases from 120 MPa to 140 MPa, an increase of 4.2% can be seen. As the packing pressure continues to increase, the stress peak begins to decrease continuously until it drops to 29.1 MPa at a packing pressure of 160 MPa.

**Figure 13:**
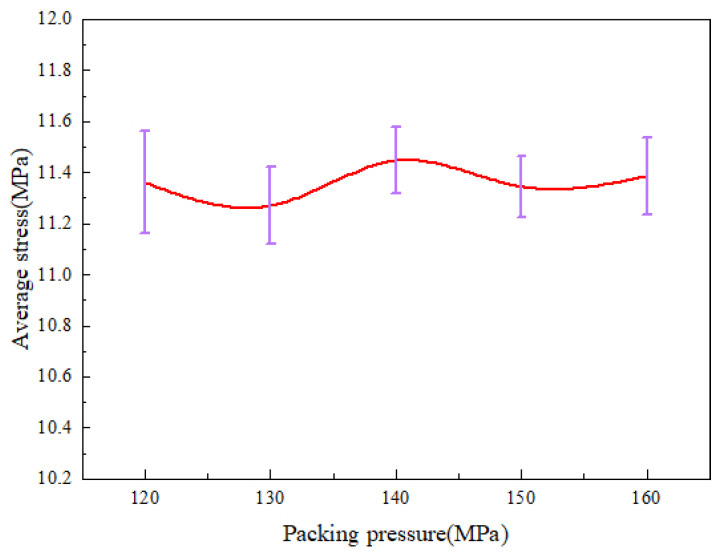
Average stress variations at different packing pressures along the centerline of the parts.

As shown in Figure 13, it can be found that the average stress changes in much are basically the same as the above. The average stress reaches the maximum at the packing pressure of 140 MPa, which is 11.45 MPa. In general, the effect of packing pressure on the residual stresses is not obvious.

**Figure 14:**
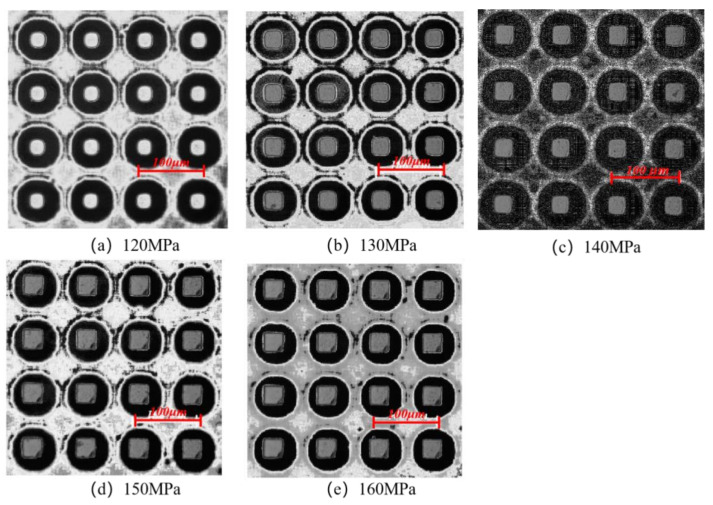
Microstructure replication at morphologies different packing pressures.

Figure 14 shows the morphologies of the micro-pillar arrays at different packing pressures in the experimental study. As the melt fills the cavity more completely with increasing packing pressure, the microstructures are also denser. In general, the degree of replication of microstructures improves with the increase in packing pressure. It can be found that the microstructures are uniform in size at a packing pressure of 130 MPa. When the packing pressure increases to 140 MPa, the microstructures become more complete, but the defects are more obvious. At the packing pressure of 150 MPa, the lower right corner of the fabricated part is warped. This is due to the fact that when the packing pressure is too high, the adhesion between the PP polymer and the Ni mold insert will be stronger, and the resistance caused by demolding will be higher. Therefore, increasing the packing pressure increases the possibility of defects.

**Figure 15:**
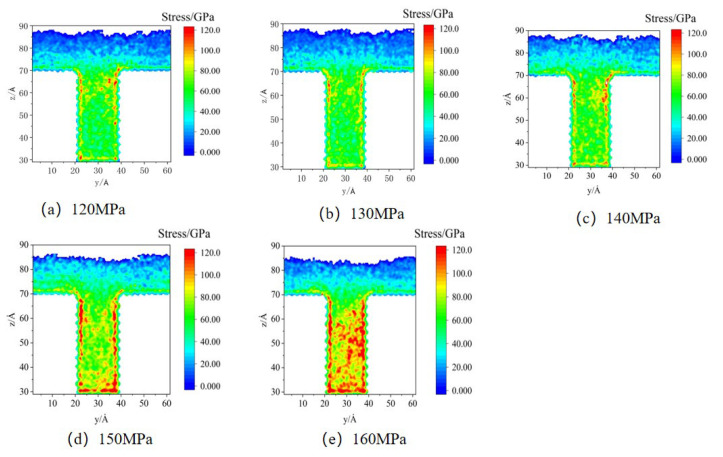
Stress distributions at different packing pressures in MD simulation.

In the simulations, different pressures (1.2–1.6 kcal/mol Å) were applied to all atoms to represent the packing pressure in order to investigate the effect on residual stress formation. The stress distributions in the cavity in the MD are shown in Figure 15. The results show that the residual stresses appear first in the side walls of the cavity. As the packing pressure increases, the stress concentration zone gradually expands to the center of the cavity and fills the whole cavity at a pressure of 160 MPa.

**Figure 16:**
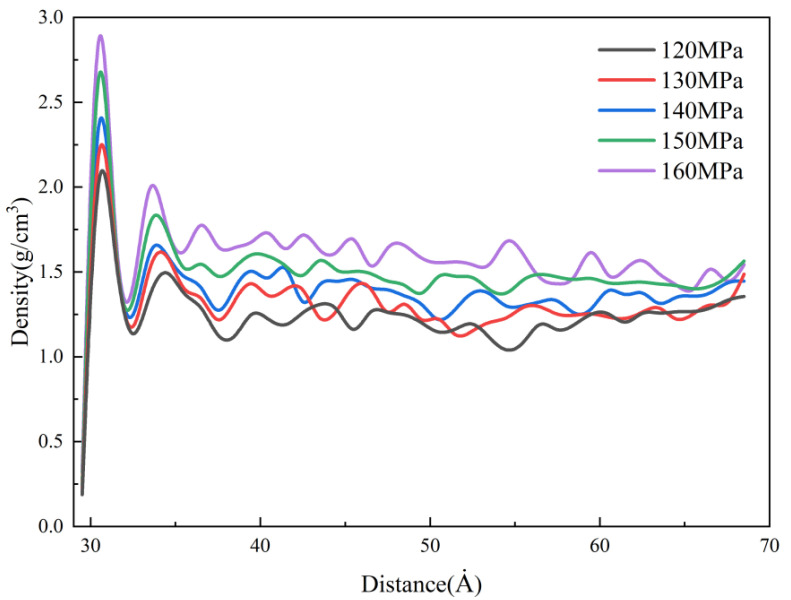
Density distributions at different packing pressures along the flow direction.

The density distributions of the cavity under different packing pressures in the MD simulation are shown in Figure 16. It can be found that the packing pressure has a significant effect on the density of nanostructures. At a packing pressure of 160 MPa, the density of the condensation layer reaches about 2.8 g/cm^3^. This is mainly due to the effect of the packing pressure, which makes the free volume of PP polymer continuously compressed, resulting in a gradual reduction of the intermolecular distance and a denser polymer, so the density is increasing. However, changing the packing pressure has almost no effect on the condensation layer’s thickness, which depends mainly on the cooling rate of the melt, so the effect of packing pressure on the thermal stresses is small. In the experiment, an increase in packing pressure will cause the melt to fill the cavity more completely, thereby improving the replication quality of microstructures. However, when the packing pressure is too high, the possibility of local defects increases.

**Figure 17:**
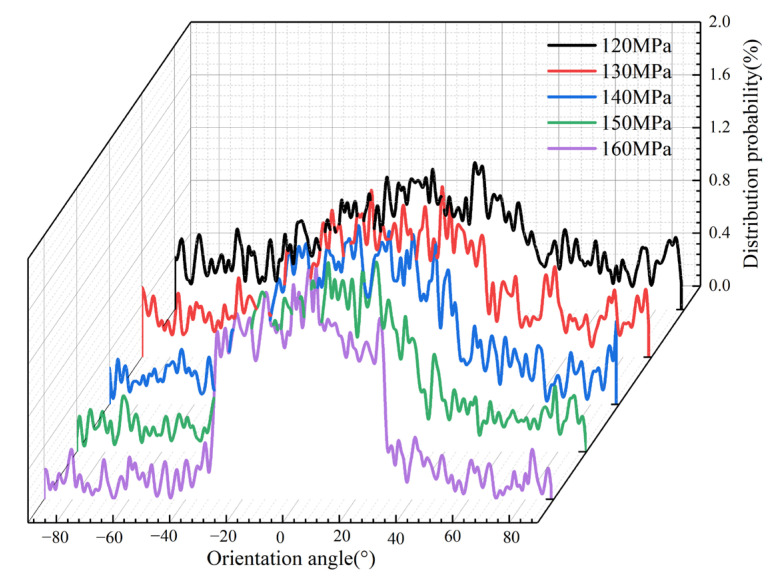
Orientation angle distributions of PP at different packing pressures along the flow direction.

The variation of the packing pressure brings different shear effects on the polymer in the cavity, which will also affect the flow state of the molecular chains. Therefore, the molecular orientation distribution curves at different packing pressure are output in the MD simulation, as shown in Figure 17. It can be seen that when the packing pressure is 120 MPa, the fluctuation range of the probability curve of the orientation angle distribution is not large. When the packing pressure exceeds 140 MPa, the distribution probability of orientation angles at −30° and 30° drastically increases continuously. On the contrary, the distribution probability of the orientation angle in the range of −30° to −90° and 30° to 90° decreases continuously with the increase in the packing pressure. When the packing pressure increases to 160 MPa, its distribution in this range is almost zero. This indicates that the increase in packing pressure forces the molecules to be more mobile, and the molecular chains are continuously oriented along the flow direction, leading to an increase in the orientation stress in the PP polymer. This is an important reason for the greater and more concentrated stresses in the cavity during the packing stage. This phenomenon indicates that the packing pressure increases the orientation stress, and therefore the adhesion between the PP polymer and the Ni mold insert will be stronger and the resistance during demolding will be higher. As a result, warpage occurs at the edges and corners during demolding, and the residual stresses in the molded parts after demolding increase significantly.

However, as the packing pressure increases, there is very little increase in residual stresses in the cavity in the experiments, while there is a large increase in residual stresses in the simulation. This is due to the fact that the cavity is not in an ideal sealing condition in the experiments, which is different from the simulation and leads to a difference in the results.

### 4.3. Effect of Melt Temperature on Residual Stresses

**Figure 18:**
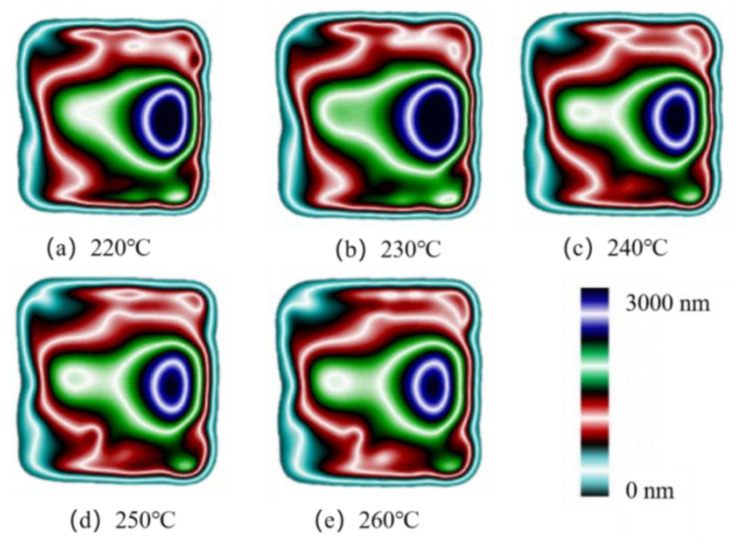
Birefringence distributions at different melt temperatures.

**Figure 19:**
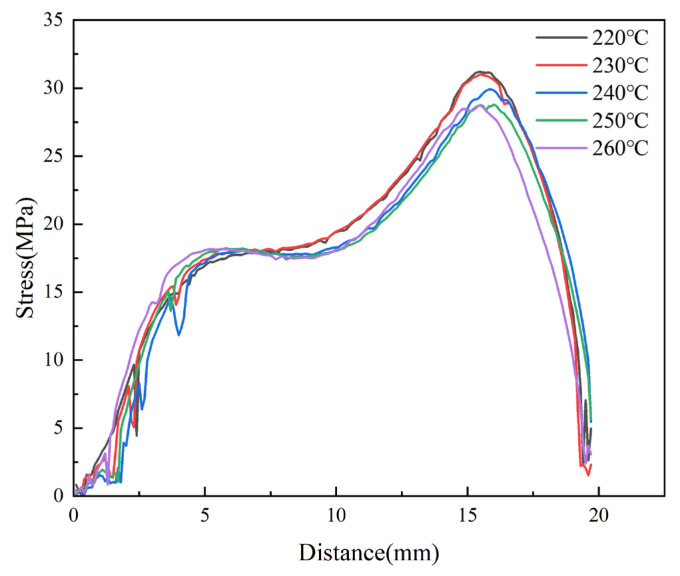
Variations of stress curves at different melt temperatures along the centerline of the parts.

**Figure 20:**
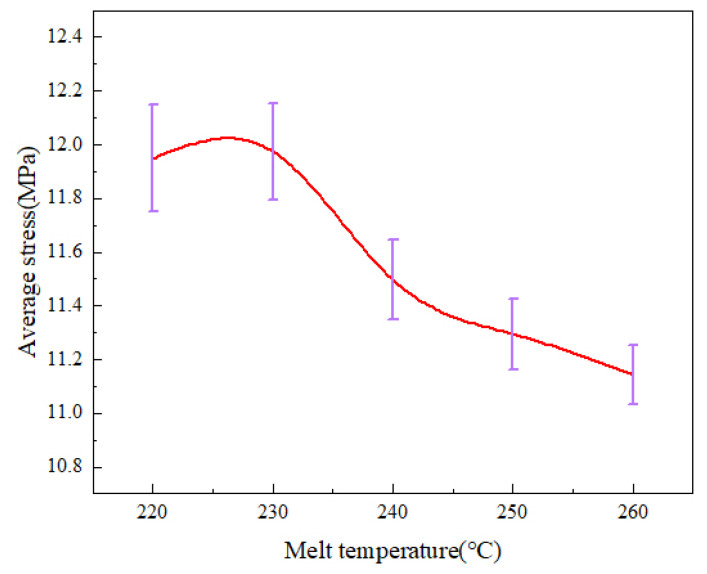
Average stress variations at different melt temperatures along the centerline of the parts.

Micro-injection molding experiments were carried out at different melt temperatures. The birefringence distributions of residual stresses in PP micro-pillar arrays are shown in Figure 18. As the melt temperature increases, the ellipse of the innermost dark blue layer, which is the maximum stress layer, keeps shrinking and the area keeps decreasing. While the green layer in the lower right corner is replaced by a red layer, the outermost light blue layer is also disappearing. Intuitively, the overall residual stresses in the parts continuously decrease as the melt temperature increases. The stress variation curves are shown in Figure 19. It can be seen that as the melt temperature increases from 220 °C to 260 °C, the stress curves essentially overlap until a distance of 5 mm from the origin, where the stress peak is obtained. However, the peak stress at a distance of 15.5 mm obviously decreases from 31.2 MPa to 28.7 MPa. Figure 20 presents the graph of the average stress lines at different melt temperatures. The average stress tends to increase slightly when the melt temperature rises from 220 °C to 230 °C, while it decreases from 11.97MPa to 11.14MPa continuously when the melt temperature increases from 230 °C to 260 °C by almost 6.9%. Therefore, within a certain range, the residual stresses inside the part can be properly reduced by increasing the melt temperature.

**Figure 21:**
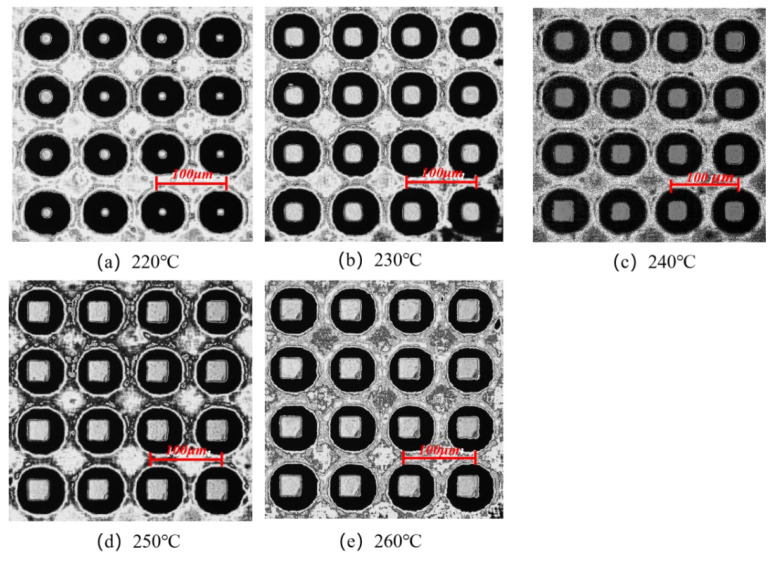
Microstructure replication morphologies at different melt temperatures.

The morphologies of the micro-pillar arrays at different melt temperatures were examined in the experimental study, as shown in Figure 21. With the increase in the melt temperature, the microstructure morphology changes from a dotted profile at 220 °C to a complete profile at 260 °C. As the melt temperature increases, the molecular chain orientation ability decreases, and the stress in the microstructure before demolding is less, which will be beneficial for the demolding stage and the replication quality will be improved. Therefore, increasing the melt temperature can not only reduce the residual stresses in the micro-pillar array after demolding to some extent but also significantly improve the molding quality of micro-pillars.

**Figure 22:**
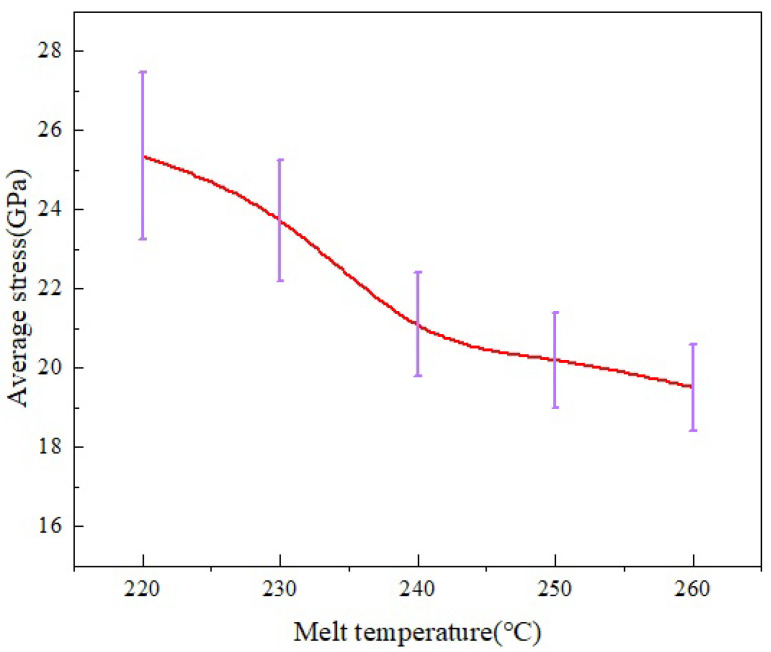
Average stress distribution at different melt temperatures in the MD simulation.

The distributions of average stress and uniformity at different melt temperatures in the MD simulation are shown in Figure 22. It can be seen that as the melt temperature increases, the overall average stress of PP shows a decreasing trend. When the melt temperature is increased from 220 °C to 260 °C, the average stress decreases by 23% and the standard deviation also decreases, indicating that the uniformity of stress distribution also improves.

**Figure 23:**
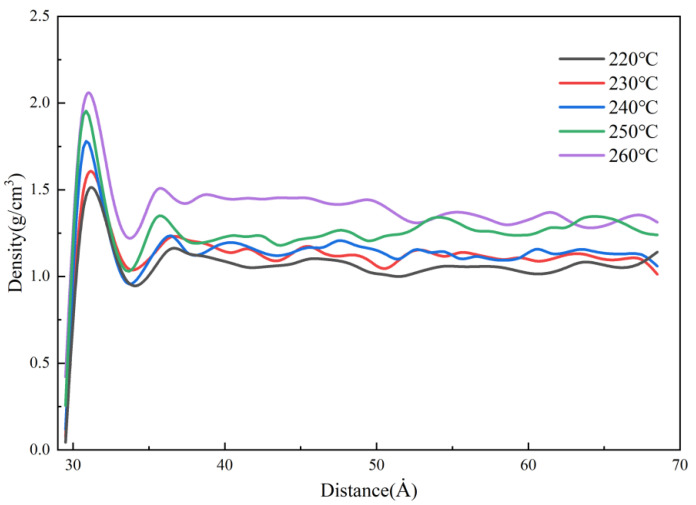
Density distribution at different melt temperatures along the flow direction.

Figure 23 shows the density distributions of the melt at different melt temperatures in the MD simulation. It can be seen that the density rises sharply and reaches a peak near the cavity wall, and the peak value increases with the increase in the melt temperature. This is because as the melt temperature increases, the temperature difference with the mold is greater, the polymer shrinks more when it contacts the wall cavity, and the density increases. Additionally, the melt temperature increases the thermal motion of the molecules, which makes the flow resistance decrease. In other words, it is good for transferring the pressure to cool and solidify more of the melt, so that the maximum density can reach 2.1 g/cm^3^ at the melt temperature of 260 °C.

**Figure 24:**
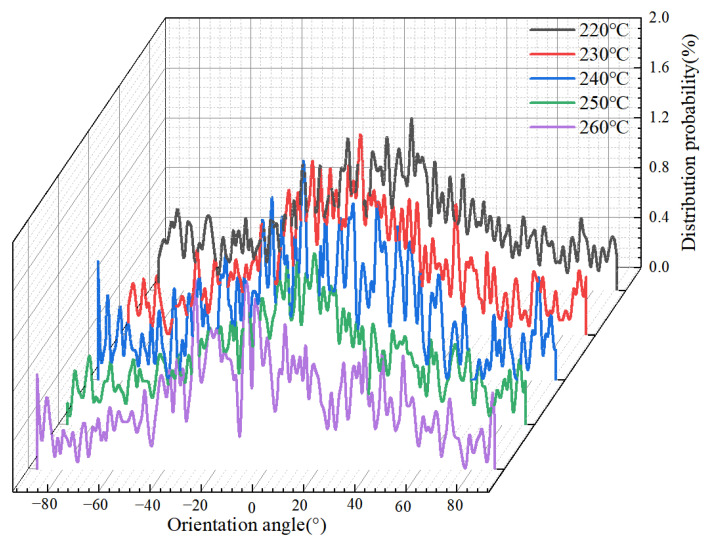
Orientation angle distributions of PP at different melt temperatures along the flow direction.

The simulation results of the orientation degree at different melt temperatures are shown in Figure 24. It can be seen that the effect of the melt temperature on the orientation degree is not obvious. When the polymer filling is completed, the overall orientation peaks around 0°, indicating that both the flow of polymer and the restriction of the cavity walls cause the orientation of the molecular chains. At the melt temperature of 220 °C, a single peak appears on the probability distribution curve of the orientation angle with a value of about 1.2%. The probability density peak of the orientation angle near 0° decreases slightly with increasing melt temperature, indicating that increasing the melt temperature to a certain extent could reduce the orientation of molecular chains along the flow direction and improve the uniformity of residual stresses distribution. This is consistent with the experimental results. In the experiments, the decrease in orientation stress leads to less stress in the microstructure before demolding, and the replication quality is accordingly improved.

## 5. Conclusions

In this study, the mechanism of the effect of the main processing parameters on residual stresses was investigated by molecular dynamics simulations and experiments. Injection molding experiments were conducted to examine the residual stresses and surface morphology of the molded micro-pillar arrays. The distribution of residual stresses, changes in melt density, and orientation angle during the injection molding process were simulated. In addition, the mechanism of the effect of temperature and pressure on residual stresses was explored.

The results of the study show that the mold temperature and the melt temperature can reduce the residual stresses and improve their uniformity of distribution. As the mold temperature and melt temperature increase, the average residual stress in the micro-pillar arrays decreases by 28.4% and 6.9% and significantly improves the replication quality of microstructures. This is due to the fact that the shrinkage of PP and the molecular chains with the increase in temperature, because of the change in cooling rate. As a result, the thermal residual stresses and molecular orientation stresses are reduced, which can effectively improve the uniformity of residual stress distribution. The average residual stress increases nonlinearly to a peak value of 11.45 MPa at a packing pressure of 140 MPa. The possibility of local defects on micro-pillar arrays increases with the increase in the packing pressure. This is because increasing the packing pressure makes the shear field of flow more intense and increases the molecular orientation stress, which results in severe residual stresses.

## Figures and Tables

**Table 1:** Average dimensions of the designed micro-pillar array, its Si master and Ni mold insert.

	Width	Depth
Designed micro-pillar array	60 μm	20 μm
Si master	60 μm	20 μm
Ni mold insert	62.5 μm	24.5 μm

**Table 2:** Material properties of polypropylene (PP).

Material	Density ρ (kg/cm^3^)	Melt Flow Rate v (g/10 min)	Heat Deflection Temperature T (°C)	Shrinkage S (%)
PP	0.9	8	88	1.4–1.8

**Table 3:** Processing parameters used in the experiments.

Processing Parameter	Values
Packing pressure (MPa)	120, 130, 140, 150, 160
Mold temperature (°C)	60, 70, 80, 90, 100
Melt temperature (°C)	220, 230, 240, 250, 260

## Data Availability

The data presented in this study are available on request from the corresponding author.
